# ExamPle: explainable deep learning framework for the prediction of plant small secreted peptides

**DOI:** 10.1093/bioinformatics/btad108

**Published:** 2023-03-10

**Authors:** Zhongshen Li, Junru Jin, Yu Wang, Wentao Long, Yuanhao Ding, Haiyan Hu, Leyi Wei

**Affiliations:** School of Software, Shandong University, Jinan 250101, China; Joint SDU-NTU Centre for Artificial Intelligence Research (C-FAIR), Shandong University, Jinan 250101, China; School of Software, Shandong University, Jinan 250101, China; Joint SDU-NTU Centre for Artificial Intelligence Research (C-FAIR), Shandong University, Jinan 250101, China; School of Software, Shandong University, Jinan 250101, China; Joint SDU-NTU Centre for Artificial Intelligence Research (C-FAIR), Shandong University, Jinan 250101, China; School of Software, Shandong University, Jinan 250101, China; Joint SDU-NTU Centre for Artificial Intelligence Research (C-FAIR), Shandong University, Jinan 250101, China; Hainan Key Laboratory for Sustainable Utilization of Tropical Bioresource, College of Tropical Crops, Hainan University, Haikou 570228, China; Hainan Key Laboratory for Sustainable Utilization of Tropical Bioresource, College of Tropical Crops, Hainan University, Haikou 570228, China; School of Software, Shandong University, Jinan 250101, China; Joint SDU-NTU Centre for Artificial Intelligence Research (C-FAIR), Shandong University, Jinan 250101, China

## Abstract

**Motivation:**

Plant Small Secreted Peptides (SSPs) play an important role in plant growth, development, and plant–microbe interactions. Therefore, the identification of SSPs is essential for revealing the functional mechanisms. Over the last few decades, machine learning-based methods have been developed, accelerating the discovery of SSPs to some extent. However, existing methods highly depend on handcrafted feature engineering, which easily ignores the latent feature representations and impacts the predictive performance.

**Results:**

Here, we propose ExamPle, a novel deep learning model using Siamese network and multi-view representation for the explainable prediction of the plant SSPs. Benchmarking comparison results show that our ExamPle performs significantly better than existing methods in the prediction of plant SSPs. Also, our model shows excellent feature extraction ability. Importantly, by utilizing *in silicomutagenesis* experiment, ExamPle can discover sequential characteristics and identify the contribution of each amino acid for the predictions. The key novel principle learned by our model is that the head region of the peptide and some specific sequential patterns are strongly associated with the SSPs’ functions. Thus, ExamPle is expected to be a useful tool for predicting plant SSPs and designing effective plant SSPs.

**Availability and implementation:**

Our codes and datasets are available at https://github.com/Johnsunnn/ExamPle.

## 1 Introduction

Plant Small Secreted Peptides (SSPs) are crucial intercellular messenger molecules that regulate a multitude of processes (Matsubayashi 2014). SSPs are usually encoded within preproteins consisting of 100–250 amino acids. These preproteins are then digested into shorter bioactive peptides (Lease and Walker 2006; Breiden and Simon 2016; de Bang et al. 2017) that exhibit physiological effects at extremely low doses, often in the nanomolar range (Murphy et al. 2012).

SSPs have become a significant class of regulatory chemicals involved in plant growth, development, and plant–microbe interactions (Czyzewicz et al. 2013; Nakaminami et al. 2018). During plant growth and development processes, most SSPs act as signaling molecules that participate in cell-to-cell communication by binding membrane receptors and coordinating responses with plant hormones (Murphy et al. 2012; Fukuda and Ohashi-Ito 2019). In terms of meristem maintenance, CLE14 and CLE40 expression have been reported to play a part in regulating meristematic activity and cell number in the root meristematic zone of the *Arabidopsis thaliana* plant (De Smet et al. 2008; Meng and Feldman 2010). Additionally, SSPs are involved in a variety of biotic stress responses in diverse plant species. For instance, a SSP named SYSTEMIN discovered in *Solanum lycopersicum* was the first wound response signaling peptide (Pearce et al. 1991; Constabel et al. 1998). When herbivores or pathogens attack plants, SYSTEMIN can activate several defense signals and pathways by interacting with SYSTEMIN RECEPTOR 1 (SYR1). These pathways and signals include the stimulation of PROTEASE INHIBITOR production as well as the enhancement of ethylene and jasmonic acid biosynthesis (Kandoth et al. 2007; Wang et al. 2018). What’s more, SSPs are crucial for other physiological processes in plants, such as embryogenesis, reproductive development, pathogen interaction, and increasing fertilizer-use effectiveness (Matsubayashi 2014; Breiden and Simon 2016). Given their diverse effects on plants, SSPs are of interest as potential strategies to improve plant performance.

In traditional biological experiments, researchers use protein mass spectrometry (MS) to discover SSPs. For example, a novel 15-aa SSP named CEP1 encoded by AT1G47485 is identified in Arabidopsis thaliana (*A. thaliana)* by liquid chromatography–mass spectrometry analysis (Ohyama et al. 2008). However, it is a difficult task to identify plant SSPs on a large scale using biological experiments because traditional biological experiments require much time and manpower. In recent years, more and more computational methods have been developed to solve various biological problems (Mrozek 2020; Li et al. 2021a,b, 2022). To address the mentioned issue above, researchers have developed some computational methods for predicting plant SSPs. To predict - SSPs containing N-terminal signal sequence (NSS), Teufel et al. (Teufel et al., 2022) proposed SignalP 6.0, a model based on protein language models for the prediction of signal peptides. Since N-terminal signal sequence usually exists in SSPs, SignalP6.0 can be used for predicting SSPs as well. The potential problem is that it might produce false positives as the NSS also often occurs in serveral types of membrane proteins (Uhlén et al. 2015).

The method mentioned above have a number of shortcomings, and there is a lack of publicly available tools for specifically predicting the plant SSPs. Importantly, these methods do not consider the analysis of the sequential patterns of the plant SSPs, which is significant for peptide downstream tasks. To address these problems, we propose **ExamPle**, a novel **Ex**plainable contrastive hybrid-view du**a**l Sia**m**ese framework for **P**lant Smal**l** S**e**creted Peptide prediction and sequential pattern discovery. Notably, with a hybrid view of peptide sequences and secondary structures, we use the Siamese network architecture and contrastive learning to automatically learn context-sensitive feature representations, sufficiently capturing discriminative information to represent the plant SSPs. Experiment results show that our model has excellent prediction performance for plant SSPs prediction and is significantly better than existing machine learning methods. Comparison between handcrafted feature encodings and our model using dimension reduction methods proves the great learning ability of our model. Furthermore, by quantatively evaluating the amino acid contribution, we demonstrate that ExamPle is capable of discovering important sequential characteristics that impact the predictive performance and identify the contribution function of each amino acid, which is bascially consistent with the statistical results. In addition, by accumulating functional differences between a variant and original sequence, we reveal that the head sequential region of the peptide and some specific sequential patterns are strongly associated with the SSPs function. Overall, ExamPle can not only provide the excellent prediction ability for plant SSPs but also give sufficient insights on effective and stable plant SSPs design.

## 2 Materials and methods

### 2.1 Dataset

*Plant SSPs dataset*: We collect positive samples of plant SSPs dataset from *MtSSPdb* (Boschiero et al. 2020). *MtSSPdb* is a comprehensive database that contains plant SSPs discovered in the model legume *Medicago truncatula*. As for negative samples, we find two non-secreted peptide families CYSTM and DVL from previous studies (Butenko et al. 2009; Xu et al. 2018); afterwards, we search for non-SSPs sequences and download them from the *Pfam* database, which is a large collection of protein families (Mistry et al. 2021). After that, we use CD-Hit (Li and Godzik, 2006) to reduce the similarity of the sequences in the dataset to 20%. Besides, datasets with balanced positive and negative samples generally benefit the training process of deep learning models. Consequently, we take the same number of positive and negative samples from the two databases. Following the above procedure, we yield the plant SSPs dataset, including 1184 plant secreted peptides and 1184 plant non-secreted peptides. Furthermore, the dataset is divided into training and testing datasets according to the proportions of 80% and 20%, respectively.

*Peptide secondary structure*: In this study, we use the PHAT web interface to generate peptide secondary structure. PHAT was proposed by Jiang et al. (2023). PHAT is a novel deep learning framework for predicting peptide secondary structures. A powerful pre-trained protein language model and a novel hypergraph multi-head attention network are utilized in PHAT, which can not only transfer semantic knowledge from large-scale proteins to peptides, learning high-latent and long-term features of peptide residues but also encode peptide residues with multi-semantic secondary structural information while capturing contextual features from consecutive regions using multi-level attention mechanisms. Besides, PHAT has an interactive, code-free, and non-programmatic web interface. We use the PHAT web interface to obtain the predicted 3-state secondary structure of peptides, and then the 3-state secondary structure information is added to the plant SSPs dataset.

### 2.2 Framework overview

**Figure 1:**
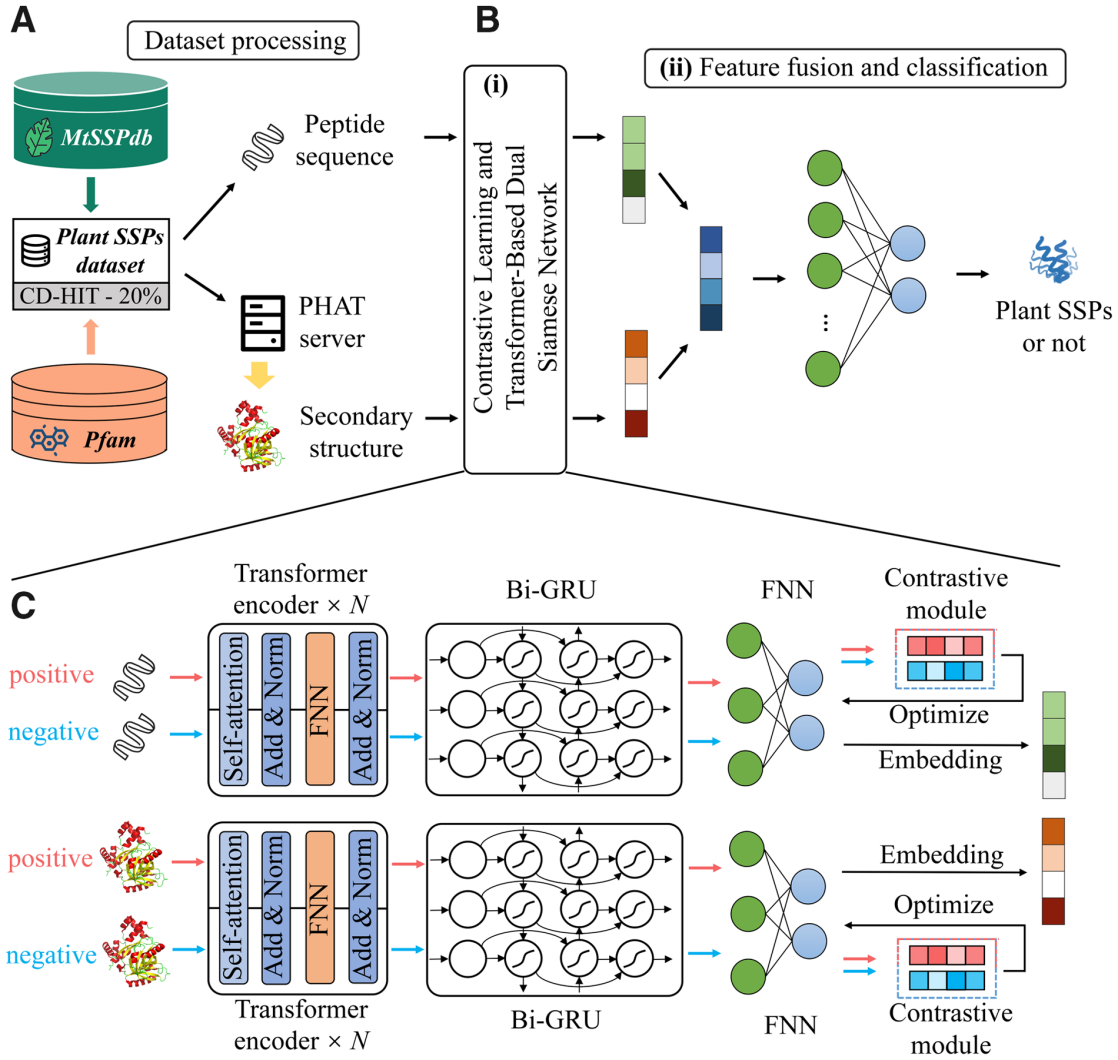
The overview of the ExamPle framework. (A) It presents how we pre-process and construct the plant SSPs dataset and how we obtain the peptide secondary structure information. (B(i)) We feed the peptide sequences and peptide secondary structure into the dual Transformer-based Siamese network to get their corresponding representations. (B(ii)) After acquiring the corresponding representations, the ExamPle can automatically fuse the two features, and then feed the fusion features to the discriminator for classification. (C) As for the peptide sequences and secondary structures, we separately use a Siamese network composed of Transformer, Bi-GRU, and FNN to extract the latent features. Meanwhile, we use the contrastive module to optimize the parameters of the network

Here, we propose a new predictive framework based on the dual Transformer and Bi-GRU architecture and Siamese network, which fuses peptide sequence and secondary structure information to predict the plant SSPs. Our framework architecture is illustrated in Fig. 1, including B(i) contrastive learning and Transformer-based dual Siamese network and B(ii) feature fusion and classification. Firstly, in B(i), we input the plant SSPs sequences and their secondary structures into the dual Transformer-based Siamese network model for feature embedding. To be specific, each Transformer-based Siamese network includes Transformer encoder, Bi-GRU (Bidirectional-Gate Recurrent Unit), fully connected neural network (FNN) and contrastive module. The two Transformer models do not share weights. Transformer encoder is used to generate context-sensitive feature representations of SSPs sequences and secondary structures. Afterward, Bi-GRU is used to capture the feature representations with long-distance information. Subsequently, features are embedded using a FNN to generate the unified representation. After that, we can obtain the distance between two SSPs feature representations. In the contrastive module, the contrastive loss is calculated, which is utilized to decrease the distance between representation vectors with the same labels and increase the distance between representation vectors with the different labels. Secondly, in B(ii), we fuse the sequence and secondary structure features learnt from B(i). Subsequently, we use a FNN as the discriminator. With a prediction score given by the discriminator, the model can determine whether a given peptide belongs to the plant SSPs. It belongs to the plant SSPs if the prediction score is higher than 0.5.

#### 2.2.1 Transformer

Transformer was proposed by Vaswani et al. (2017), which was initially applied for machine translation tasks and had become the state-of-the-art model in a large number of Natural Language Processing (NLP) tasks. Compared to the previous methods based on protein energy (Mrozek et al. 2007; 2009; Malysiak-Mrozek and Mrozek 2011), AlphaFold achieves great success by using Transformer (Jumper et al. 2021). So, in this work, we adopt Transformer as our model’s backbone. In the Transformer part of our model, we use the original version of Transformer. Transformer is composed of the encoding module and the decoding module. The encoding module comprised of a series of encoders used to extract the features of inputs. Additionally, the decoding module consists of a series of decoders that are used for generating representations. Here, we only extract features using a Transformer encoder. The Transformer encoder layer is illustrated in Fig. 1.

Aiming to encode the positional information, the Transformer has a positional vector added to it, which is implemented to each embedding of inputs using sine and cosine functions. In other words, the output $X_{i,\mathrm{embed}}$ of the embedding layer is


(1) Xi,embed=EmbeddingXi+PosEmbeddingXi 

Afterward, $X_{i,\mathrm{embed}}$ is fed to a Multi-headed Self-Attention (MSA) block and a Multilayer Perceptron (MLP) block. The layer-normalization (LN) is applied before each block and residual connections are applied after each block. The output of the first encoder layer ${X_{i,\mathrm{output}}}^{1}$ is


(2) Xi,output1=MSALNXi,embed1+Xi,embed1 

(3) Xi,output1=MLPLNXi,output1+Xi,output1 

Then, ${X_{i,\mathrm{output}}}^{1}$ is fed into the second encoder layer, generating the output denoted as ${X_{i,\mathrm{output}}}^{2}$, which is the same in the following encoder layers. Suppose there are n encoder layers in the Transformer encoding module, the output of the last layer is ${X_{i,\mathrm{output}}}^{n}$. After above operation, we can gain a context-dependent embedded representation. The Transformer utilizes the multi-head attention mechanism, which captures not only the local information in a window of a fixed area of the sequence but also the global information of the sequence, explaining why the representation is reliant on context. With the ability of learning context-sensitive embedding representation, this approach can not only resolve the word ambiguity issue in the realm of NLP successfully but also improve token representation in peptide sequences significantly. In addition, the later GRU model also benefits from this context-sensitive embedding representation.

#### 2.2.2 Gated recurrent unit

Gated recurrent unit (GRU) was proposed by Cho *et al.* (2014). It aims to make each recurrent unit to capture dependencies of different time scales adaptively. The architecture of GRU has been simplified compared to Long Short-Term Memory (LSTM) (Hochreiter and Schmidhuber 1997) while keeping the same effectiveness as LSTM. The GRU has gating units that modulate the flow of information inside the unit similarly to the LSTM unit, but without having a separate memory cell. The input gate, forget gate, and output gate—three essential parts of an LSTM cell—are transformed into two gates in GRU: update gate ($z_{t}$) and reset gate ($r_{t}$). GRU combines the unit state and output into one state $h_{t}$. For each element (time step t) in the input sequence, each layer computes using the functions as follows: where $W_{z}$, $W_{r}$, and W are weights of GRU; $h_{t}$ is the hidden state at time step t; $h_{t-1}$ is the hidden state of the layer at time t-1 or the initial hidden state at time 0; σ is the sigmoid function; and ∘ is the Hadamard product. In a multilayer Bi-GRU, the input $x_{t}^{\left(l\right)}$ of the l-th layer (l ≥ 2) is the hidden state $h_{t}^{\left(l-1\right)}$ of the previous layer. After passing through K layers, we can obtain the final flattened hidden state output $h^{K}$ ($h^{K}=[h_{1}^{K},h_{2}^{K},h_{3}^{K},\ldots ,h_{L}^{K}]$) of the layer K.


(4) zt=σWz·ht-1,xt 

(5) rt=σWr·ht-1,xt 

(6) ht∼=tanhW·rt∘ht-1,xt 

(7) h=1-zt∘ht-1+zt∘ht∼ 

#### 2.2.3 Siamese network and contrastive module

The Siamese network can be used to quantify the similarity of two sequences. For example, aiming to finding matching and non-matching pairs of images, Melekhov et al. (2016) obtained the feature vectors with convolutional neural networks which were learnt from labeled examples of matching and non-matching image pairs by using a contrastive loss function in a Siamese network architecture. Li and Zhang (2019) proposed a Siamese network called SiamVGG that combined a convolutional neural network backbone and a cross-correlation operator, taking advantage of the features from exemplary images for more accurate object tracking. To be specific, given two input vectors $v_{1}$ and $v_{2}$, they are input into the same modules. The network maps the inputs to the same feature space. Afterwards, the network computes the similarity of the two inputs using distance measuring function, denoted as $D(v_{1},v_{2})$. Here, we use Euclidean distance $D(v_{1},v_{2})$:


(8) Dv1,v2=∥v1-v2∥2 

After gaining the hidden state output $h^{K}$, we use a FNN to map it to the same feature space. By doing that, we can map an input vector of a sequence $X_{i}$ to a representation vector $v_{i}$:


(9) vi=MLPBi-GRUTransformerEncoderXi 

Aiming to decrease the distance between representation vectors of peptide sequences with the same label and increase the distance between representation vectors of peptide sequences with the different label, we use contrastive loss (Hadsell et al. 2006) $L_{1}$ as the loss function: where Y=0 if sequences $X_{1}$ and $X_{2}$ have the same label and Y=1 if they are different. And m>0 is a margin. If the distance between $v_{1}$ and $v_{2}$ is further than m, we are not going to optimize; Otherwise, if the distance between $v_{1}$ and $v_{2}$ is smaller than m, the distance between them will be further. That is, the margin defines a radius around v, and only dissimilar pairs whose distance falls inside this range contribute to the loss function.


(10) L1v1,v2,Y=121-YDv1,v22+12Ymax⁡0, m-Dv1,v22 

In this way, we can use gradient descent algorithm to optimize the parameters of the network and generate unified representation for each inputs pair. Also, we need a discriminator D(v) to map the representation vector to the class outputs. Consequently, cross-entropy loss is utilized as one of the loss functions in our model:


(11) L2v,Y=-YlogDv-1-Ylog⁡1-Dv 

To be mentioned, if the FNN and the discriminator are connected directly, the parameters will be updated jointly, which will result in the catastrophic forgetting (McCloskey and Cohen 1989). In order to avoid this circumstance, we freeze the parameters of the previous layers, only updating the parameters of the discriminator when training the model. Consequently, the comprehensive loss function of our model is where $Y_{1}$ and $Y_{2}$ are the labels of $v_{1}$ and $v_{2}$ and $Y_{1}=1$ if $v_{1}$ is positive, else $Y_{1}=0$.


(12) Lv1,v2,Y,Y1,Y2=L1v1,v2,Y+L2v1,Y1+L2v2,Y2 

### 2.3 Performance measures

Refer to previous works, we use following classification metrics to evaluate our model: accuracy (ACC), specificity (SP), sensitivity (SE), and Matthew correlation coefficient (MCC). The formulas of these metrics are as follows:


(13) ACC=TP+TNTP+TN+FP+FN 

(14) SP=TNTN+FP 

(15) SE=TPTP+FN 

(16) MCC=TP×TN+FP×FNTP+FPTP+FNTN+FNTN+FP 

In the formulas, TP is the number of true-positive samples, FP is the number of false-positive samples, TN is the number of true-negative samples, and FN is the number of false-negative samples. Accuracy is the ratio of the correctly classified samples to the total samples, which is the most intuitive performance measurement. Specificity measures the proportion of the negative samples which are classified correctly, and Sensitivity measures the proportion of the positive samples which are classified correctly. MCC takes into account positives and negatives, so that it is generally regarded as a balanced metric to measure the overall performance of a model.

## 3 Results

### 3.1 Performance comparison of ExamPle with other methods on benchmark datasets

**Figure 2:**
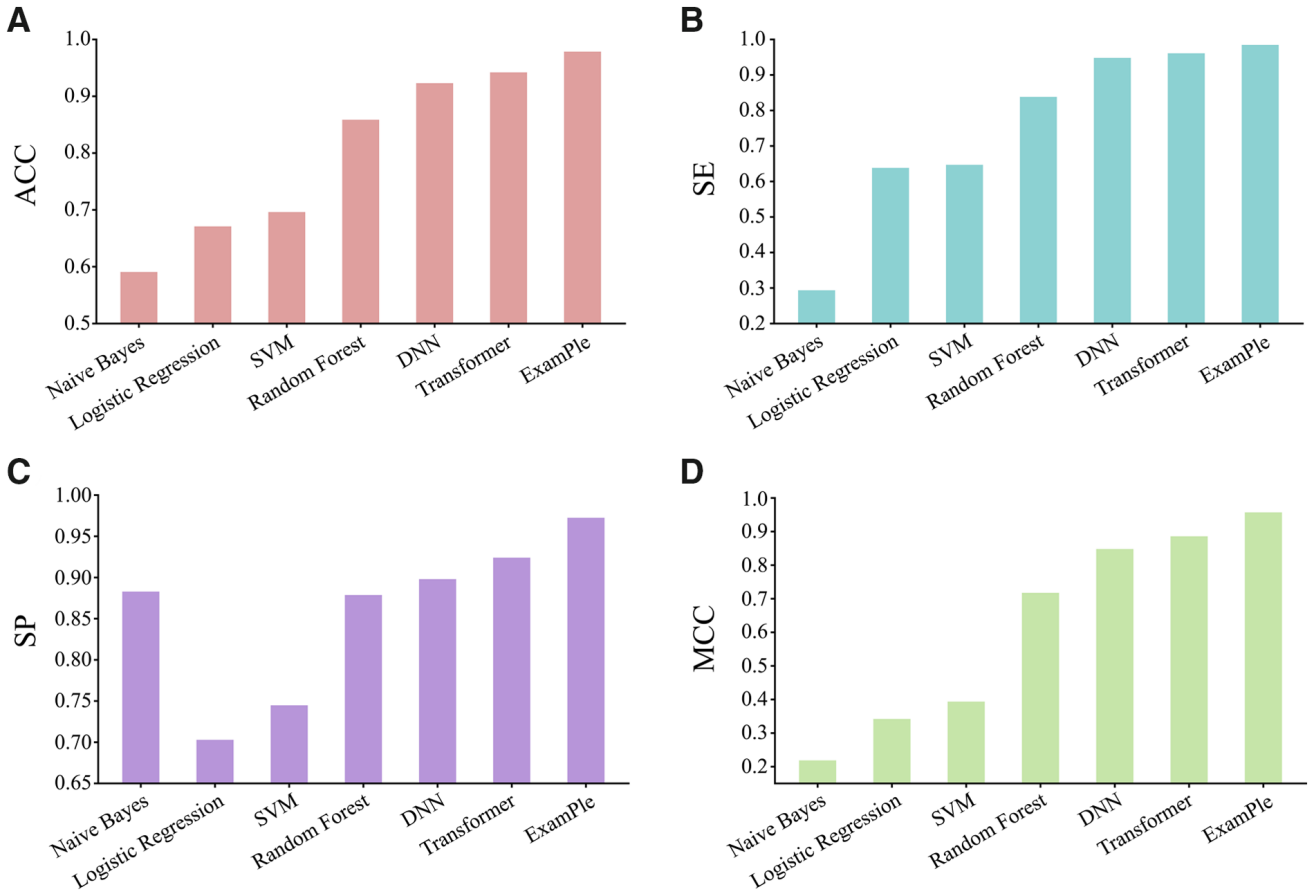
Performance comparison between ExamPle, several classic machine learning models, and deep learning models. (A–D) respectively represent the ACC, SE, SP, and MCC values of our proposed ExamPle and other models, including Naïve Bayes, Logistic Regression, SVM, Random Forest, DNN, and Transformer

To evaluate the performance of ExamPle, we adopted several classic comparative models including Naive Bayes (Rish 2001), Logistic Regression (Hosmer et al. 2013), SVM (Support Vector Machine) (Cortes and Vapnik 1995), Random Forest (Breiman 2001), DNN (Deep Neural Networks) (Montavon et al. 2018), and standard Transformer considering the lack of directly predicting SSPs in previous work. For a fair comparison, we used training set for model training and evaluated these models in independent dataset within the same setting. For better reproducibility of the comparative experiment, the architectures of DNN and Transformer are implemented by DeepBIO (Wang et al. 2022) server. As illustrated in Fig. 2, ExamPle outperforms all of the comparative models in terms of ACC, SE, SP, and MCC metrics. For instance, the performance in ACC of ExamPle increases by 3.65% compared to Transformer and increases by 11.99% compared to Random Forest; the performance in MCC increases by 10.91% compared to DNN and increases by 56.35% compared to SVM. In conclusion, these results demonstrate that the ability to effectively predict plant SSPs of ExamPle is better than that of the majority of machine learning and deep learning approaches.

### 3.2 The feature representation of ExamPle is better than handcrafted feature encodings

In order to verify the effectiveness of the feature representation learned by ExamPle, we compared the performance of different handcrafted feature methods with our model. With the help of iLearnPlus (Chen et al. 2018, 2020, 2021), we chose five feature encoding methods, including Amino Acid Composition (AAC) (Spackman et al. 1958), CTD Composition (CTDC), CTD Distribution (CTDD), CTD Transition (CTDT) (Dubchak et al. 1999), and Conjoint Triad (CTriad) (Shen et al. 2007). The detailed information on these handcrafted feature encodings can be found in Supplementary Table S1. Then, we separately feed five handcrafted feature encoding methods into the classification layer of our model for training and testing.

**Figure 3:**
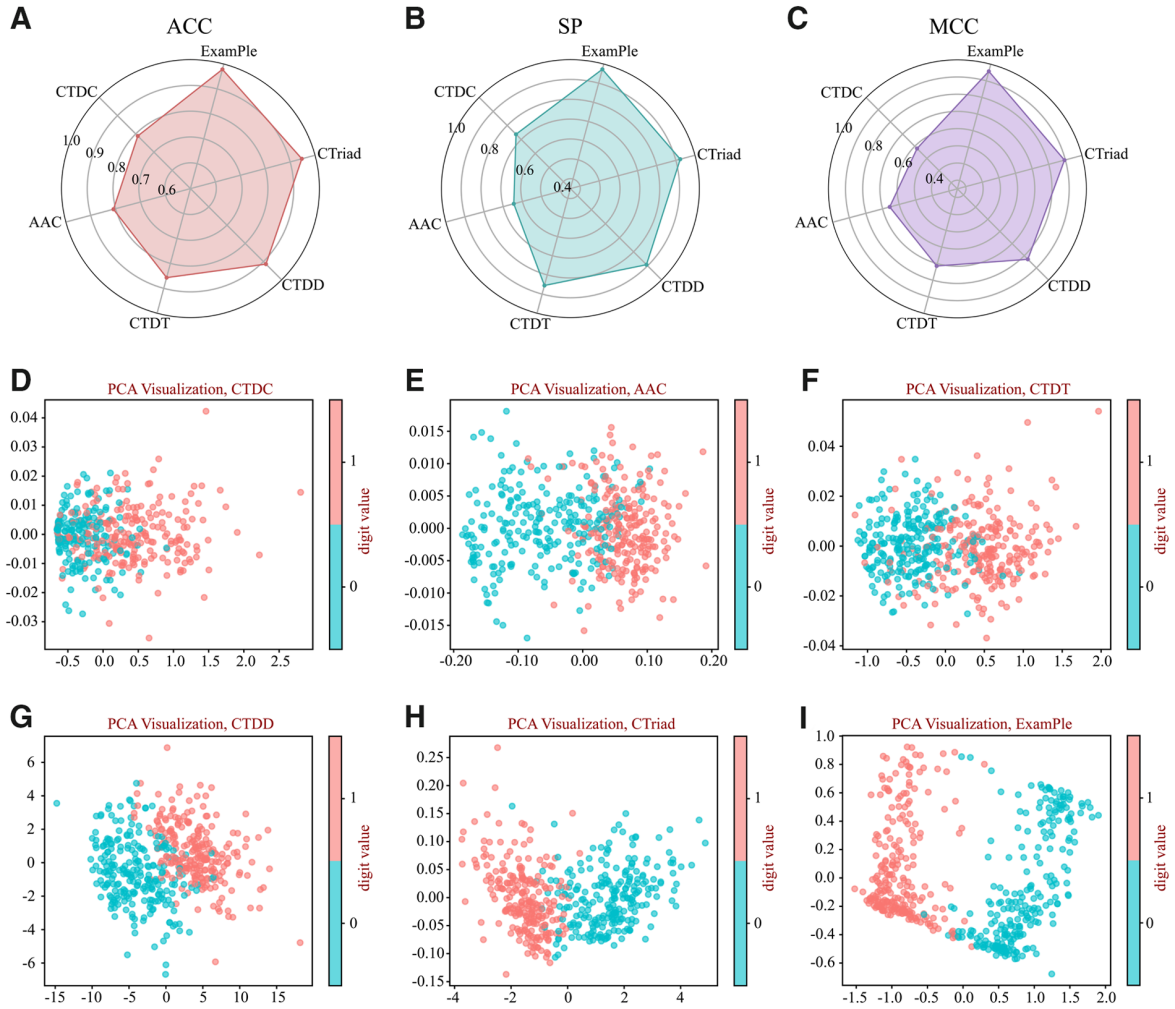
Performance comparison between the feature representation of ExamPle and other five handcrafted feature encoding methods. (A–C) respectively represent the ACC, SP, and MCC values of the feature representation of ExamPle and other handcrafted feature encoding methods, including CTDC, AAC, CTDT, CTDD, and CTriad. (D–I) Feature visualization results of ExamPle and other five handcrafted feature encoding methods are shown

As shown in Fig. 3, the CTriad encoding method reaches 94.67% on ACC, while the CTDC encoding method reaches 78.86%. Nevertheless, the representation embedded by ExamPle is superior to all other handcrafted feature encoding methods. Especially when it comes to the performance of MCC, our model is 37.57% higher than CTDC and 24.28% higher than CTDT. To further validate the performance of ExamPle, we visualized the feature representations embedded by five handcrafted feature encoding methods as well as our model using dimension reducting. As illustrated in Fig. 3(D-I) , we can more intuitively see that the representation encoded by our model can be classified better than any other five handcrafted feature representations. In summary, with excellent adaptability and robustness, our model can learn a more powerful feature representation compared to other handcrafted feature encoding methods.

### 3.3 The visualization of feature representations during the training process

**Figure 4:**
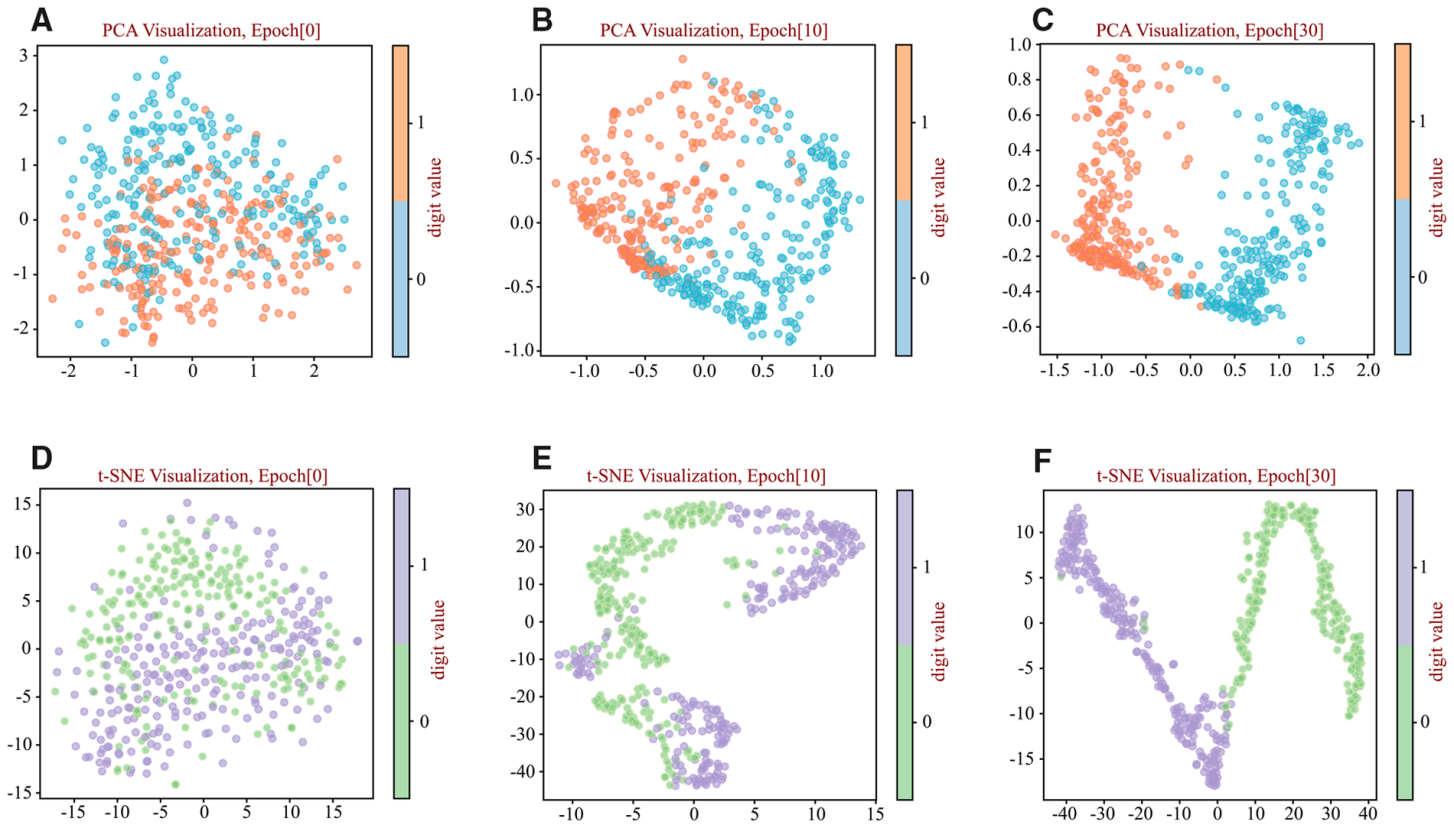
Visualization of samples in the test dataset by PCA and t-SNE. Epoch[x] in the title of each subfigure denotes the corresponding training epoch of the model. 0 and 1 respectively represent the non-SSPs and the SSPs. (A–C) PCA visualization images at different epoch, and (D–F) show t-SNE visualization images at different epoch are shown

It is rarely clear how the deep learning model works during the training process. For a more transparent understanding, we reduced our model’s latent space to a 2D space using principal component analysis (PCA) (Abdi and Williams 2010) and t-distributed Stochastic Neighbor Embedding (t-SNE) (Van der Maaten and Hinton 2008) to verify the efficiency of ExamPle. As shown in Fig. 4, at the beginning of the training procedure, negative and positive samples are mixed together. Subsequently, as the number of training iterations increases, the samples are gradually divided into two groups, indicating our model can learn sufficient information through back propagation optimization.

### 3.4 Exploring the sequential characteristic of plant SSPs by using ExamPle

**Figure 5:**
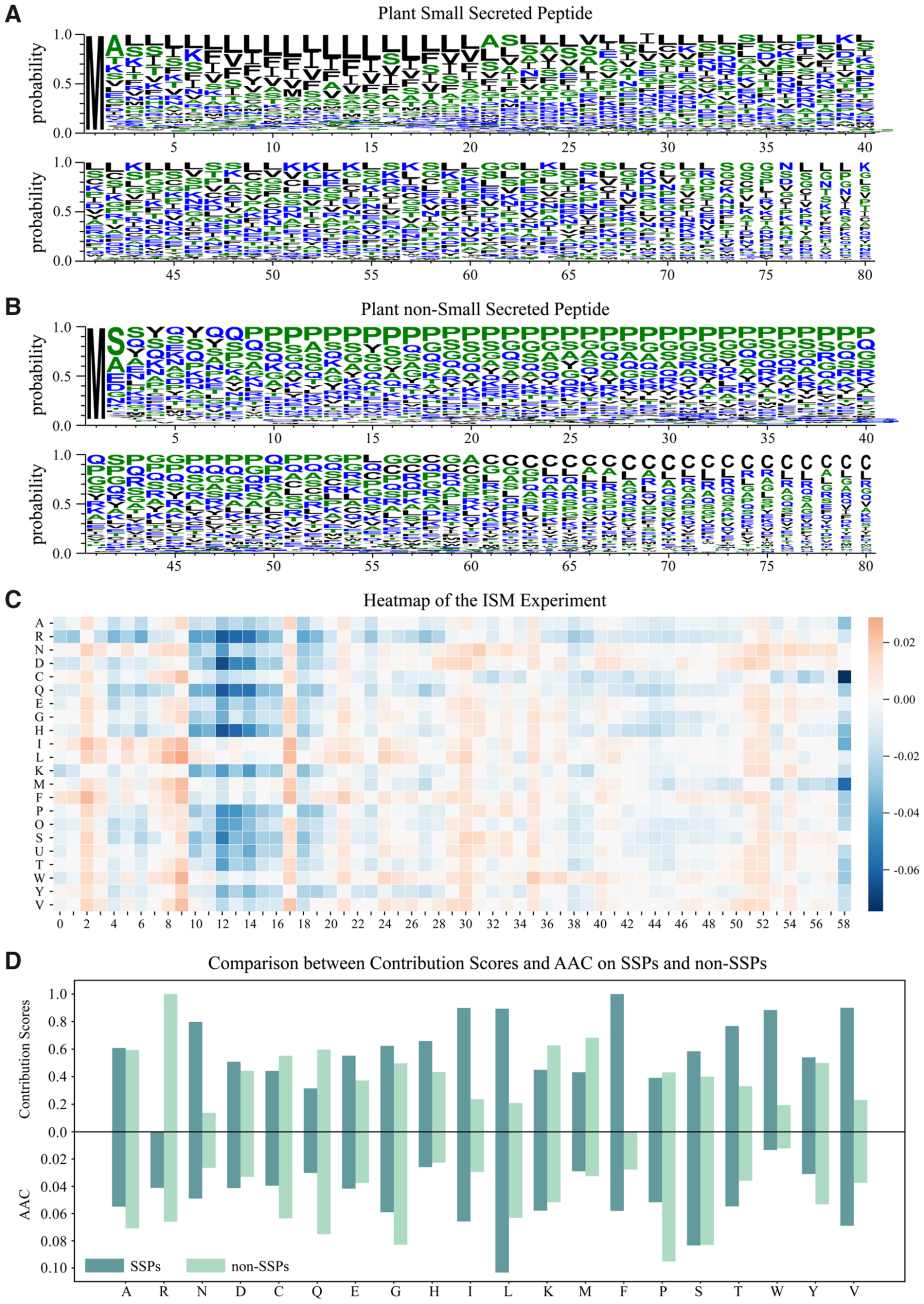
The results of exploring the sequential characteristic of plant SSPs by using our ExamPle. (A) and (B) respectively represents the probability distribution statistics of the plant SSPs and the plant non-SSPs. (C) The heat map showing the result of the ISM experiment. The positive value represents a higher prediction score for the sequence when replaced with a certain amino acid, and the negative value represents a lower prediction score. (D) The comparison results between the contribution scores and amino acid composition (AAC) of each amino acid on SSPs and non-SSPs. The contribution scores are the sum of each amino acid score on each peptide obtained from the ISM experiment

Given the excellent performance of our complex neural network architecture, we are targeting further exploration of the sequential characteristics of plant SSPs from a computation perspective. To this end, we performed in silico mutagenesis (ISM) interpretation method for every peptide in our dataset and mutated each reference amino acid of a peptide into other nineteen alternative amino acids to evaluate the predicted probability change. Moreover, we conducted amino acid statistical probability for each position on SSPs dataset and the non-SSPs dataset respectively in Fig. 5A and B as a reference to our analysis.

Here, we select one 59-bp-length sequence as the sample to generate the heat map in Fig. 5C, in which the positive value represents a higher prediction score for the sequence when replaced with a certain amino acid and the negative value represents a lower prediction score. This heat map easily reveals that the amino acids Q (*Glutamine*), and P (*Proline*) have a significant inhibitory effect on the prediction score. To be specific, in the position between 10 and 16, when the amino acid is mutated with Q or P, the prediction score is significantly reduced. The possible reason for this is that Q and P are frequently present in the non-SSPs and are present less frequently in the SSPs, as illustrated in the Fig. 5A and B. So, when recognizing Q and P in the sequence, the model tends to consider the sequence as the non-SSPs. Particularly, C (*L-Cysteine*) is frequently present in the tail of the non-SSPs. And observations from Fig. 5C implied the amino acid C significantly reduced the predicted score in the tail region. Besides, the amino acids I (*l-isoleucine*), L (*Leucine*), and F (*Phenylalanine*) have a stimulative effect on the prediction score in most positions of the sequence. The possible reason for this is that, as shown in Fig. 5A, I, L, and F appear frequently in the SSPs sequences while appearing rarely in the non-SSPs sequences. When recognizing I, L, and F in the sequence, the model tends to consider the sequence as SSPs.

Then, to make the results more statistically meaningful, we computed the contribution of each amino acid on the whole dataset. For each peptide of SSPs and non-SSPs, we calculated amino acid scores by accumulating each position score from the ISM results. In detail, we can sum the score of one row in Fig. 5C and get twenty value each representing the contribution score of twenty amino acid in this 59- bp-length sequence. Given the amino acid contribution score of each peptide, we estimated the comprehensive amino acid contribution score on SSP and non-SSP respectively by adding them up. The results of contribution of different amino acids in the SSPs dataset and non-SSPs dataset are shown in Fig. 5D. In addition, we utilized the Amino Acid Composition (AAC) as a statistical analysis reference. As can be seen in Fig. 5D, for the vast majority of amino acids, the relative contribution of each amino acid to the prediction score in the SSPs or non-SSPs is consistent with the relative AAC of this amino acid in the SSPs or non-SSPs. In short, for a particular amino acid, if it has a higher AAC in the SSPs dataset than in the non-SSPs, the contribution to the prediction scores will be likely to be higher in the SSPs than in the non-SSPs. Besides, we can see that the AAC of amino acid G (*Glycine*) in the SSPs is smaller than that in the non-SSPs, but our model considers that G contributes more to the prediction score in the SSPs, showing our model can mine features that cannot be counted by traditional statistical methods. To sum up, these experiments indicate that our model can mine the sequential characteristics of SSPs.

### 3.5 The interpretable analysis on region importance by using ExamPle

**Figure 6:**
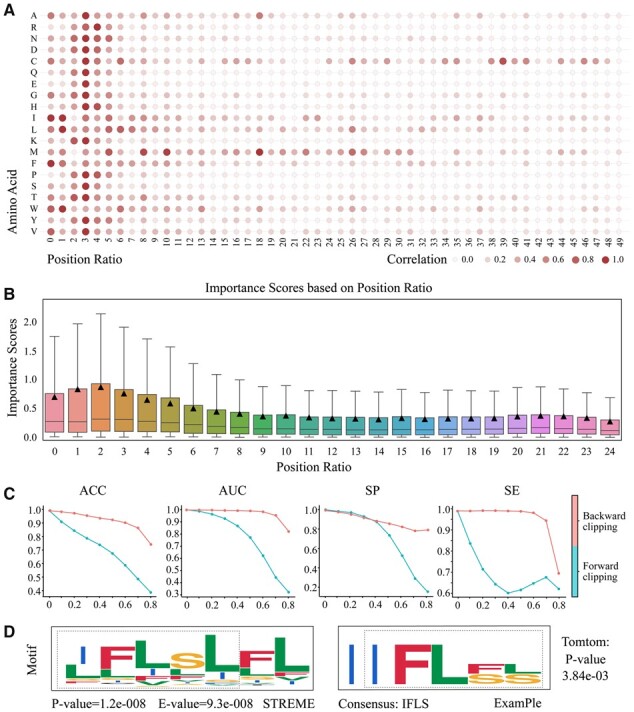
The interpretable analysis on region importance by using our ExamPle. (A) A complete correlation map between amino acid and position ratio on the whole dataset using the absolute value obtained from the ISM experiment is shown. (B) A boxplot of the importance scores based on position ratio is shown. (C) The result of the clipping experiment is shown. The forward clipping means we cut the sequence on the head region and conserve the tail region. The backward clipping means we cut the sequence on the tail region and conserve the head region. (D) The comparison between the motif searched by the conventional tool STREME and the motif extracted from our model is shown

We next seek to understand the connection between the functional impact and the sequence region. To this end, regardless of the positive or negative effect of one position, we utilized the absolute value subtracted from the ISM experiment as the importance scores to evaluate how the contextual region affects the plant SSPs’ function. To unify all the sequences into one fixed length, we split the sequence into 50 parts. Here, each part is referred as a position ratio. In Fig. 6A, we present a complete correlation map between amino acid and position ratio in the whole dataset. The colors are deeper, the importance scores are higher. For example, amino acid M (*Methionine*) has a more functional impact on the central region, while amino acid C (*Cysteine*) has a more functional impact in the tail region, which are both meaningful points to be noticed in the peptide design. Next, to further intuitively illustrate which region is more important, we divided each peptide in the dataset into 25 parts and accumulated the amino acid scores to get the comprehensive score of the position. In Fig. 6B, it is obvious that there is an increasing trend on important scores in the head region and a slow decline in the central region. Between the position ratios 1 and 3, the median of the importance scores is twice as high as that of the position ratios behind 9.

To better verify the phenomenon in Fig. 6B, we conducted a clipping experiment where we clipped the sequence from the forward direction and backward direction respectively, and fed the clipped sequence into the model to investigate whether the clipped sequence can conserve the original function. As can be seen in Fig. 6C, the backward clipping performance is very robust, and the accuracy remains above 0.9 until the clipping ratio increases to 0.5. However, the metric of forward clipping declines rapidly once the clipping operation starts, and the ACC of the whole dataset is even lower than 0.5 when the clipping ratio exceeds 0.7. Interestingly, the metric SP measuring the accuracy of negative cases maintains the same level at the beginning of the clipping, which can sufficiently demonstrate that negative sequences do not rely on the head region and may be related to the central region.

After we utilize importance scores to intuitively find which region is important for the peptide function, it is very natural to explore the motif of this region from a computation perspective. In Fig. 6D, we discovered one basic motif based on the important scores and compared it with the motif searched by a conventional tool—STREME (Bailey 2021). As can be seen, our learned motif (highlighted with a gray-color window) closely resembles the STREME’s motif. Then, we utilize TOMTOM (Bailey et al. 2009) to quantitatively calculate the motif similarity measured by the *P*-value. The lower *P*-value indicates a higher degree of motif consistency. Fig. 6D demonstrates that the motif we select is highly similar to the STREME’s motif, indicating that the conserved sequential pattern can be discovered by our model. Taken together, these experiment results suggest that our model ExamPle has the potential to be a new tool for sequential pattern discovery and may pave the way for stable and effective plant SSPs design.

## 4 Conclusion

In this study, we present a novel explainable contrastive hybrid-view dual Siamese framework ExamPle for predicting the plant SSPs and discovering the sequential pattern of the SSPs. The experiment results demonstrate that the Siamese framework with secondary structure information can learn discriminative feature information and effectively enhance the performance of our model for the plant SSPs prediction. Besides, by adopting dimension reduction methods, the visualization of latent space clearly shows the great learning ability of our model and outperforms traditional handcrafted feature engineering. Utilizing ISM interpretation methods, ExamPle can discover sequence characteristics and identify the contribution function of each amino acid, which are mostly consistent with the statistical results. In addition, by accumulating functional differences between the variant and original sequences, the key novel principle learned by our model is that the head region in peptides and some specific sequential patterns are strongly associated with the SSP function. Thus, our well-trained model ExamPle can not only provide the prediction for plant SSPs but also pave the way for effective and stable plant SSPs design.

## Supplementary Material

btad108_Supplementary_Data

## Data Availability

The authors declare that the datasets and related codes are available at https://github.com/Johnsunnn/ExamPle.
