# Supplementary material for: ExamPle: explainable deep learning framework for the prediction of plant small secreted peptides
Source: Bioinformatics. 2023 Mar 10;39(3):btad108. doi: 10.1093/bioinformatics/btad108 (PMC10027287; doi:10.1093/bioinformatics/btad108)
Supplement: btad108_Supplementary_Data [file btad108_supplementary_data.docx]

Supplementary file

**Table S1. The detailed introduction of each handcrafted feature encodings**

| **Handcrafted feature encodings** | **Detailed introduction** |
| --- | --- |
| Amino Acid Composition (AAC) | Amino acid composition is the percentage of standard amino acids; it has a fixed length of 20 features. |
| CTD Composition (CTDC) | For the hydrophobicity attribute (HP), the three numbers represent C-the percent compositions of polar, neutral, and hydrophobic residues in the protein. |
| CTD Distribution (CTDD) | The first number of T is the percent frequency with which a polar residue followed by a neutral or a neutral residue by a polar residue. The second number is the frequency of a polar residue followed by a hydrophobic residue or a hydrophobic residue followed by a polar residue. The third number corresponds to the cases where a neutral residue is followed by a hydrophobic residue or a hydrophobic residue is followed by a neutral residue. |
| CTD Transition (CTDT) | The D descriptor has five numbers for each of the three residue types (neutral, polar, and hydrophobic). |
| Conjoint Triad (CTriad) | In this method, each amino acid sequence is represented by a vector space consisting of descriptors of Amino acid. To describe the properties of sequence and reduce dimensions, the 20 amino acid were grouped into seven classes according to their dipoles and volumes of the side chains. |

**Table S2. Performance comparison between ExamPle and other machine learning or deep learning models.**

| Model | ACC | SE | SP | MCC |
| --- | --- | --- | --- | --- |
| Naive Bayes | 0.5907 | 0.2936 | 0.8828 | 0.2187 |
| Logistic Regression | 0.6709 | 0.6383 | 0.7029 | 0.3420 |
| SVM | 0.6962 | 0.6468 | 0.7448 | 0.3936 |
| Random Forest | 0.8586 | 0.8383 | 0.8787 | 0.7177 |
| DNN | 0.9230 | 0.9480 | 0.8980 | 0.8480 |
| Transformer | 0.9420 | 0.9610 | 0.9240 | 0.8860 |
| **ExamPle(this study)** | **0.9785** | **0.9847** | **0.9724** | **0.9571** |

**Table S3. Performance comparison between the embedding of ExamPle and other five handcrafted feature encoding methods.**

| Model | ACC | SE | SP | MCC |
| --- | --- | --- | --- | --- |
| CTDC | 0.7886 | 0.8410 | 0.7372 | 0.5814 |
| AAC | 0.8089 | 0.9760 | 0.6452 | 0.657 |
| CTDT | 0.8571 | 0.8590 | 0.8552 | 0.7143 |
| CTDD | 0.9137 | 0.9359 | 0.8920 | 0.8295 |
| CTriad | 0.9467 | 0.971 | 0.9230 | 0.8946 |
| **ExamPle(this study)** | **0.9785** | **0.9847** | **0.9724** | **0.9571** |
